# Reproducibility of coronal plane alignment of the knee (CPAK) using tibia-first restricted inverse kinematic alignment

**DOI:** 10.1007/s00402-025-05878-w

**Published:** 2025-04-28

**Authors:** Amer Hallak, Ittai Shichman, Itay Ashkenazi, Amal Khoury, Yaniv Warschawski, Aviram Gold, Nimrod Snir

**Affiliations:** 1 Tel Aviv Sourasky medical Center, Tel Aviv-Yafo, Israel; 2 Tel Aviv University, Tel Aviv, Israel

**Keywords:** Total knee arthroplasty, Restricted inverse kinematic alignment, Coronal plane alignment of the knee

## Abstract

**Introduction:**

The Coronal Plane Alignment of the Knee (CPAK) classification categorizes knee phenotypes based on constitutional limb alignment and joint line obliquity (JLO). Restricted Inverse Kinematic Alignment (RIKA) is a surgical philosophy that incorporates calculated perioperative parameters to achieve patient-specific alignment. This study investigated the reproducibility of restoring preoperative CPAK phenotypes via the tibia-first RIKA concept in total knee arthroplasty (TKA).

**Methods:**

This prospective study assessed 129 patients who underwent RIKA TKA using an imageless navigation robotic-assisted arm with a single implant design for primary osteoarthritis between January 2022 and December 2023. Preoperative and postoperative angles for the lateral distal femoral angle (LDFA) and medial proximal tibial angle (MPTA) were measured from full-length standing anteroposterior X-ray imaging. Arithmetic HKA (aHKA) was defined as MPTA - LDFA, and JLO was outlined as MPTA + LDFA to classify each knee into one of nine CPAK phenotypes. CPAK boundaries for neutral aHKA and JLO were 0° ± 2° and 180° ± 3°, respectively.

**Results:**

The mean pre- and postoperative aHKA were − 2.73° (SD ± 4.9°) vs. -2.83° (SD ± 3.0°), respectively. The most common preoperative CPAK phenotypes were I (*n* = 74, 42.5%) and II (*n* = 40, 23.0%). Among patients with preoperative type I phenotype, 39.2% (29/74) preserved their composition postoperatively, while 48.6% (36/74) converted to type IV. Of 40 Type II knees, 65.0% (26/40) preserved alignment, with 25.0% (10/40) shifting to type V. In preoperative types IV and V, 75% (12/16) and 88.5% (23/26) preserved their phenotypes, respectively. Valgus categories (III, VI, IX) were neutralized into types II and V. CPAK types VII, VIII, and IV were rare throughout.

**Conclusion:**

The use of tibia-first RIKA achieved adequate preservation of the native coronal alignment postoperatively. These findings suggest that balancing a knee using tibia-first approach with the use of imageless navigation robotic-assisted systems is a valid tool for surgeons who seek contemplating RIKA TKA.

## Introduction

Over the upcoming years, the number of total knee arthroplasty (TKA) procedures is only expected to rise [1]. However, although the majority of patients experience clinical and functional improvement [2], a noteworthy 11–25% of patients still report dissatisfaction following TKA [3]. To address this, recent research has focused on identifying factors associated with enhanced patient satisfaction following TKA. Notably, one of the most extensively studied methods involves optimizing native knee alignment [4]. Specifically, modernized classifications and surgical approaches for acquiring that aim to improve clinical outcomes and patient satisfaction [5–7].

The patient-specific coronal plane alignment of the knee (CPAK) is a classification system first outlined by MacDessi et al. [8] which incorporates constitutional alignments to categorize the osteoarthritic knee into nine categories. It is based upon independent femoral and tibial coronal alignment parameters such as the arithmetic hip-knee-ankle angle (aHKA) and joint line obliquity (JLO). Using this model, neutral coronal alignment and horizontal JLO are targeted for all patients when using the mechanical alignment (MA) approach for TKA [9]. However, there is limited understanding of the extent, direction, and consequences of using this alignment method. This is evident in an analysis by Sappey-Marinier et al. [10], where 42% of MA TKAs failed to attain targeted alignments, with 18% returning to their pre-arthritic CPAK phenotype. Conversely, kinematic and functional alignment approaches are aimed at restoring the preoperative CPAK phenotypes [11]. Recent literature has emphasized the importance of incorporating functional knee kinematics and soft tissue behavior under load to provide dynamic framework in evaluating alignment strategies in TKA [12]. Applying such strategies has demonstrated favorable restoration of constitutional alignment [13], which is associated with favorable TKA clinical outcomes [14, 15].

The restricted inverse kinematic alignment (RIKA) is a modern coronal alignment technique which incorporates preoperative and intraoperative parameters that preserve patients’ native alignment to reach a tailored outcome [4]. This method relies on tibia-first gap balancing to restore tibial JLO, by equal medial and lateral tibial resections are performed, compensating for cartilage and bone loss [16]. However, to date, there is limited data regarding the efficacy of RIKA in restoring patients’ preoperative CPAK phenotype. A recent study by Graichen et al. [17] demonstrated that tibia-first, gap-balanced, patient-specific alignment effectively restores bony phenotypes and JLO in the majority of varus and neutrally aligned knees. Building upon these findings, the primary aim of this study was to investigate the reproducibility of CPAK phenotypes utilizing RIKA technique with imageless robotic assistance, while further incorporating intraoperative functional frameworks to comprehensively analyze alignment restoration. Secondary aims were to describe the distribution of CPAK phenotypes in our population. We hypothesized that aHKA and JLO would be preserved in varus knees, yet valgus knees shifting to neutral alignments postoperatively.

## Methods

**Fig. 1:**
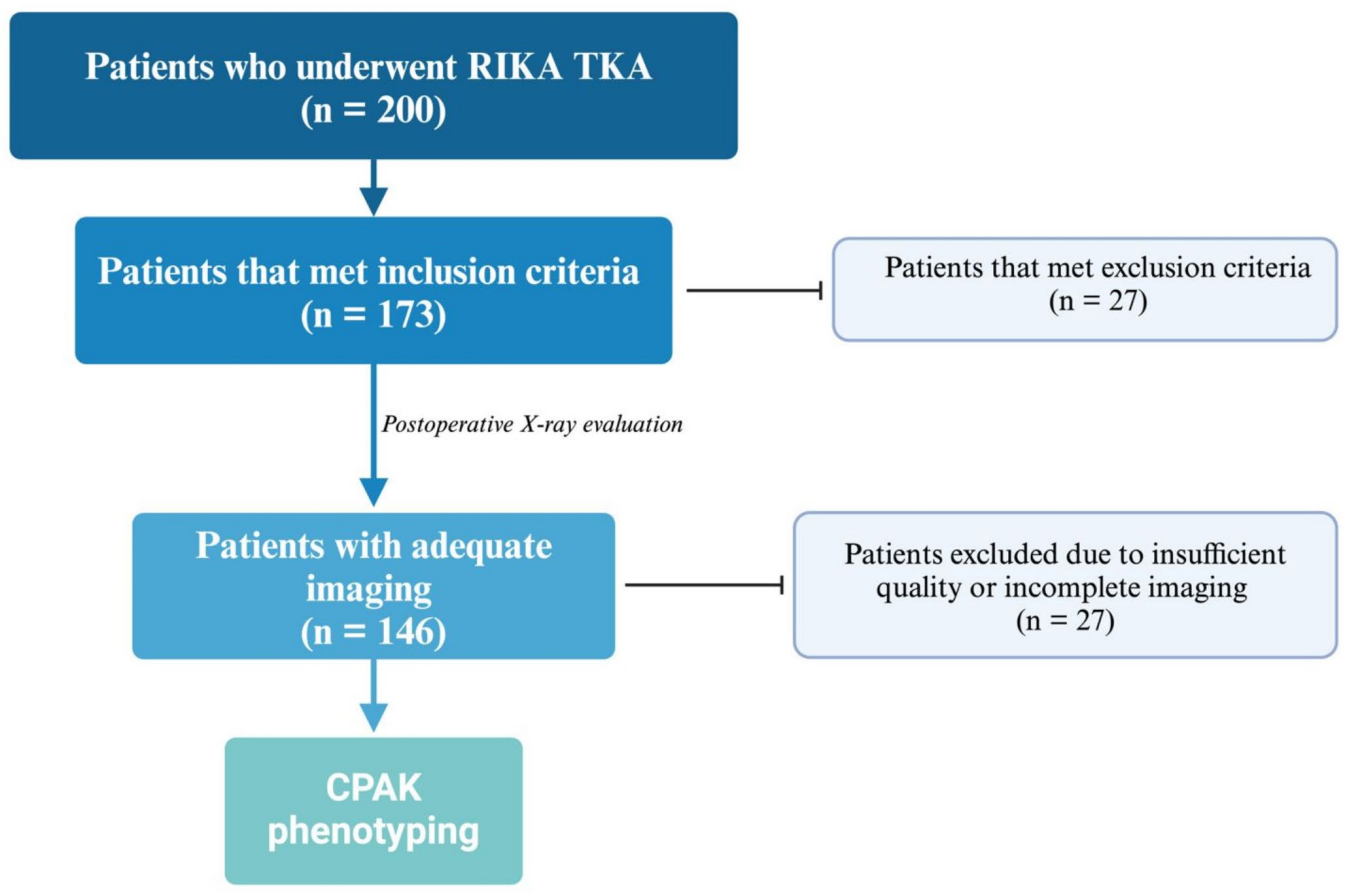
Demonstration of patient selection throughout the study

Following approval from our institutional review board, electronic medical records (EMR) were reviewed to identify patients who underwent RIKA imageless robotic-assisted TKA (RIKA TKA) between January 2022 and December 2023. Only patients who underwent an elective primary RIKA TKA as treatment for primary osteoarthritis (OA) were included in the study. Patients with secondary OA (i.e., post-traumatic, post-infectious, or inflammatory etiologies), pathologic knee lesions, and those who did not complete pre- and postoperative standing anterior-posterior (AP) weight-bearing full-body low-dose imaging were excluded. EMRs were reviewed to collect demographic data, including age, sex, follow-up time and laterality. Of the 200 patients who underwent RIKA TKA, a total of 173 (86.5%) patients met our inclusion criteria and were included in the study, with the remaining cases being lost to follow-up. A formal a priori sample size calculation was not performed, as this study is a prospective observational cohort that includes all eligible patients during the defined study period. Patients were then categorized into one of the nine CPAK groups based on the degree of pre- and post-operative knee coronal alignments. These groups were subsequently analyzed and compared [Figure 1].

### Radiographic assessment and measurements

All patients included in the study have completed pre- and post-operative standing AP weight-bearing full-body low-dose imaging. During the radiographic assessment, appropriate rotational knee position (patella facing forward) was confirmed before final acquisition of the full-limb AP radiographs. All radiographic images were digitally obtained using a picture archiving and communication system (PACS). Those of inadequate quality or incomplete imaging where consequently excluded thereafter (*n* = 27 patients).

**Fig. 2:**
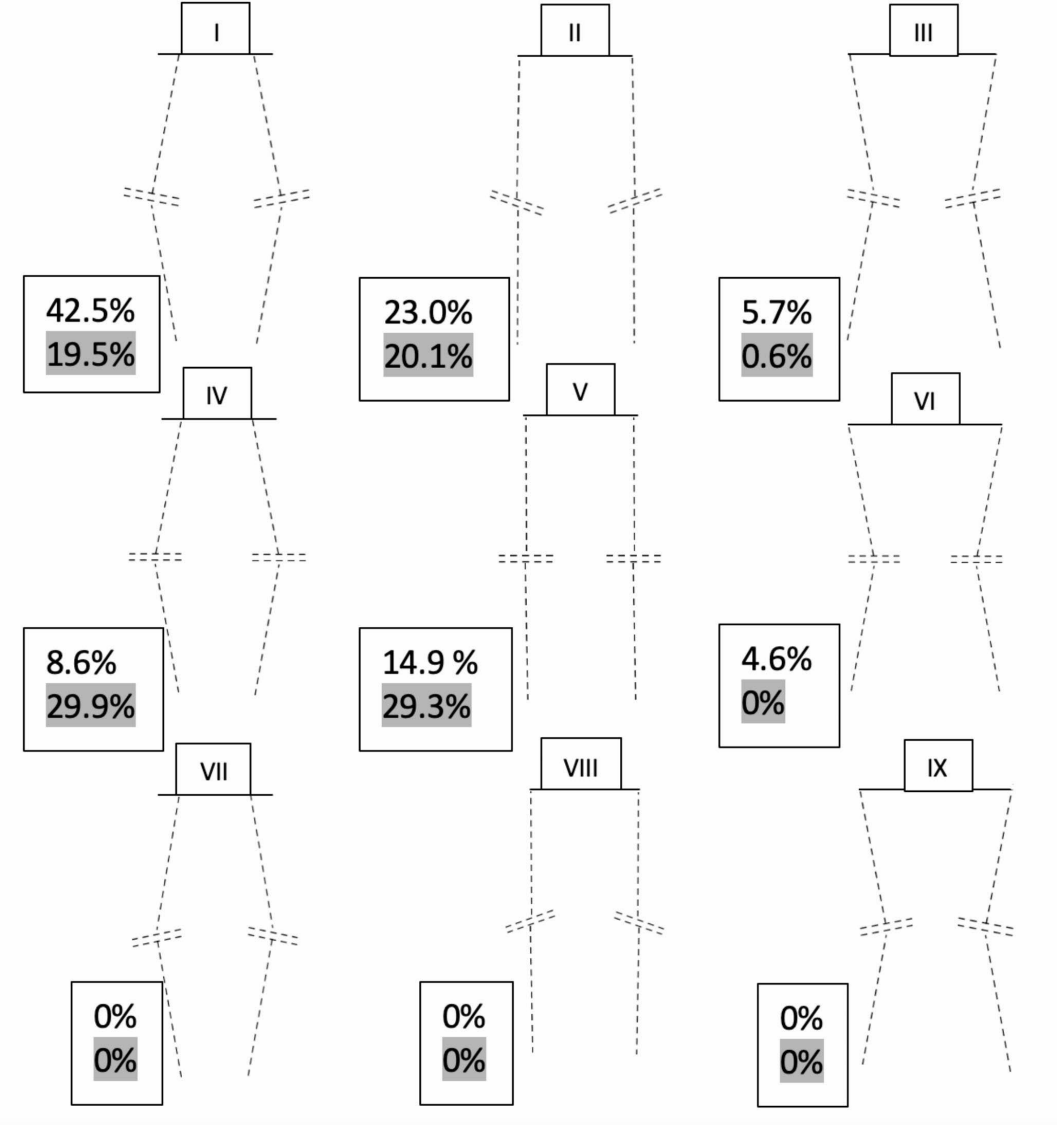
Percentage of patients according to their coronal plane alignment of the knee (CPAK) classification. Clear values represent preoperative status, darkened values represent postoperative

Analysis was done on the PACS using the Visage 7 Imaging Software (Visage Imaging Inc, San Diego, CA, USA). All radiographs were reviewed by one of two fellowship-trained surgeons from the author group (IS and NS). The radiographic measurements encompassed several aspects: lateral distal femoral angle (LDFA) and medial proximal tibial angle (MPTA) were measured from full-length standing anteroposterior X-ray imaging. Arithmetic HKA (aHKA) was defined as *MPTA - LDFA* and JLO was outlined as MPTA + LDFA to classify each knee into one of nine CPAK phenotypes described by MacDessi et al. [8]: Type I– varus distal apex; Type II– neutral distal apex; Type III– valgus distal apex; Type IV– varus neutral apex; Type V– neutral neutral apex; Type VI– valgus neutral apex; Type VII– varus proximal apex; Type VIII– neutral proximal apex; Type IX– valgus proximal apex [Figure 2].

CPAK boundaries for neutral aHKA and JLO were 0° ± 2° and 180° ± 3°, respectively [8].

Inter-observer reliability was tested using intra-class correlation coefficient (ICC) with a 2-way random effects model, assuming single measurements and absolute agreement. Sample size for reliability testing was calculated with an ICC target value of 0.8 and a 95% confidence interval width of 0.2. A minimum number of inter-observer reliability for the 2 raters was 20 by Bonnett’s approximation [18]. Thus, a subset of 20 radiographs were read by both the surgeons. ICC for measurements of LDFA was 0.87 (0.79–0.90) and 0.83 (0.79–0.88) for MPTA and was deemed excellent.

### Surgical data and technique

All surgeries were performed by one of three adult reconstruction fellowship trained orthopaedic surgeons with 7–20 years of post-fellowship experience.

The RIKA TKA of all cases were performed by using the VELYS Robotic-Assisted imageless navigation system (Depuy-Synthes, Warsaw, IN). Medial parapatellar approach was utilized for all cases included in the study. Following joint exposure, Optical trackers were affixed to the tibia and femur, and anatomic landmarks were identified to construct anatomic coordinate systems. Following the assessment of the preoperative knee kinematics, surgeons planned their resection angles intraoperatively using the system’s software, thus providing secondary insight on functional parameters on knee alignment. The target resection alignments were recorded, including the femoral sagittal, coronal and internal-external rotation angles, the tibial sagittal and coronal angles, as well as the thicknesses of the distal and posterior femoral resections and the tibial resection. ATTUNE (Depuy-Synthes, Warsaw, IN) posterior stabilized cemented knee system implants were used in all cases.

Tibia-first inverse kinematic alignment (iKA): This technique is a tibia-first gap balancing, with the aim to restore the native tibial joint line. Minimal adjustments are implemented on the femoral component position from the patient’s native femoral anatomy, utilized in the goal of achieving a balanced knee in extension while maintaining the natural lateral laxity in flexion. In every instance, iKA was conducted mirroring the technique outlined by Murgier et al. and Winnock de Grave et al. [4, 19]. Tibial registration involved digitizing the medial and lateral resection depths according to the study by Murgier et al. [19] utilizing the mid-coronal line of the lateral tibial plateau and a point marked on the tidemark of the medial plateau where cartilage wear measures approximately 2 mm. Femoral anatomy was registered using a 3D morphometric model [20]. An initial kinematic assessment was conducted to determine the range of motion under manual manipulation. Subsequently, the navigation system was used to plan tibial resection, aiming to restore the native joint line in the coronal plane, while considering cartilage wear as described by Murgier et al. [19] and restricting resection to 5° varus and 3° valgus from the mechanical axis. Tibial resection was performed accordingly using the robotic cutting saw and validated using the navigation system validation pointer. A mechanical joint tensioner was then introduced into the joint space to collect laxity data through the range of motion prior to secondary balance assessment. Data was then utilized as input for the intraoperative predictive gap-planning software, which virtually positioned the femoral component, providing a postoperative gap prediction throughout the range of motion. Femoral resections were planned to achieve stability and rectangular mediolateral gaps in extension, while permitting some lateral laxity as the knee transitions into flexion, restricting distal femoral valgus to 3° valgus and 6° varus from the mechanical axis using the predictive gap-planning software. Final laxity was determined using the implanted tibial insert thickness. Mediolateral balance was characterized as the disparity between lateral and medial laxity. All subsequent measurements were made intraoperatively and recorded into the robotic system user-interface.

### Data analysis

Pre- and postoperative comparisons were made for measured radiographic parameters. Continuous numeric variables were expressed as mean ± standard deviation (SD) and range to summarize the data distribution. Microsoft Excel built-in functions were utilized for the extraction and visualization of relevant graphs and scatter plots.

## Results

Of the total of 173 patients that satisfied the inclusion criteria, 119 were female (68.8%). The average age of the cohort was 70 years (SD ± 8.9). Surgeries were performed on left knees in 88/174 (50.9%) of cases. The average BMI of the entire cohort was 30.5 (SD ± 5.8). Postoperative imaging at follow-up averaged at 8.1 months (range, 7.4–9.2) postoperatively.

The mean pre- and post-operative aHKA were − 2.73° (SD ± 4.9°) vs. -2.83° (SD ± 3.0°), respectively. The LDFA and MPTA averaged at 88.6° (SD ± 2.4°) and 85.9° (SD ± 3.4°) preoperatively, which respectively shifted to 90.1° (SD ± 2.1°) and 87.2° (SD ± 1.7°) following surgery.

**Fig. 3:**
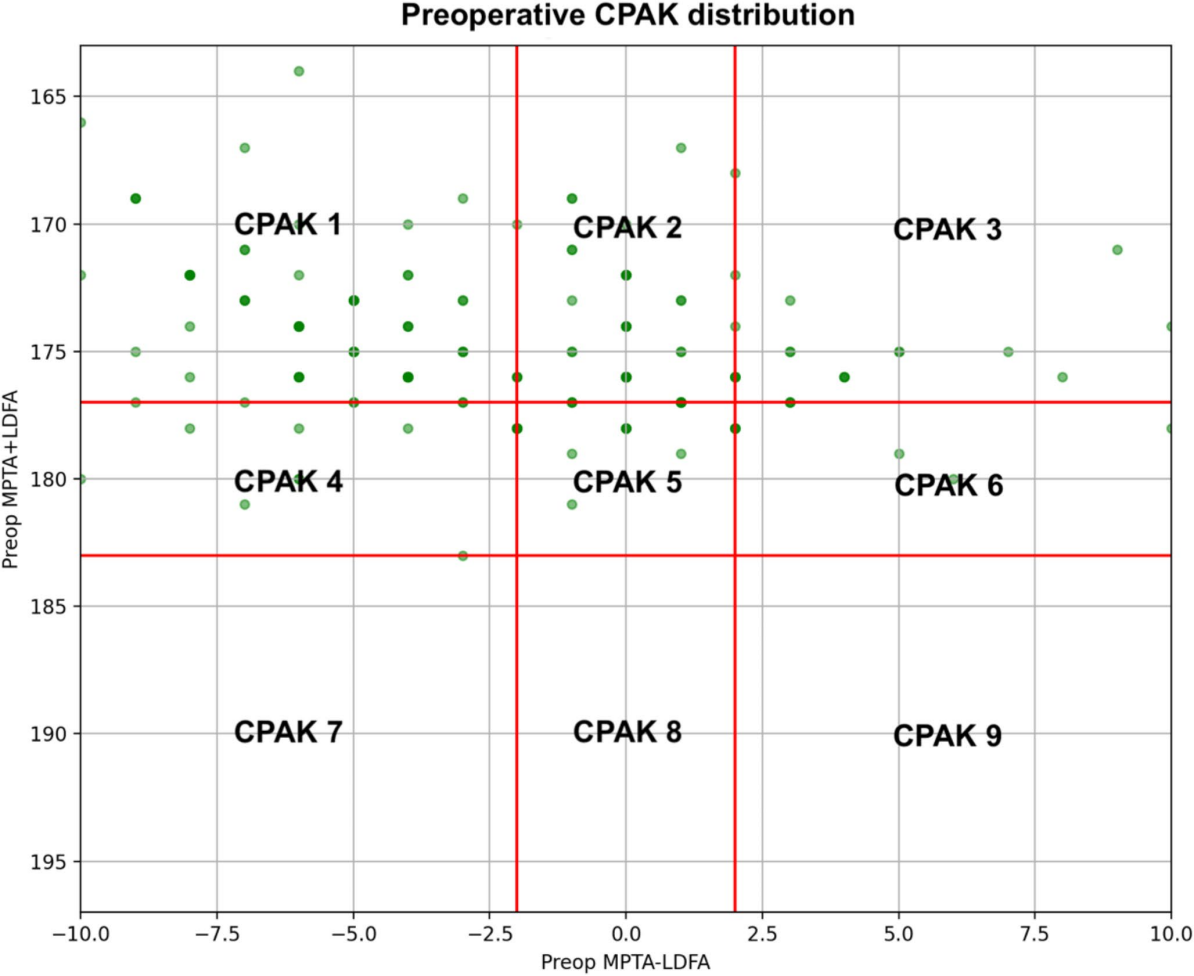
Distribution of patients according to their coronal plane alignment of the knee (CPAK) preoperatively

**Fig. 4:**
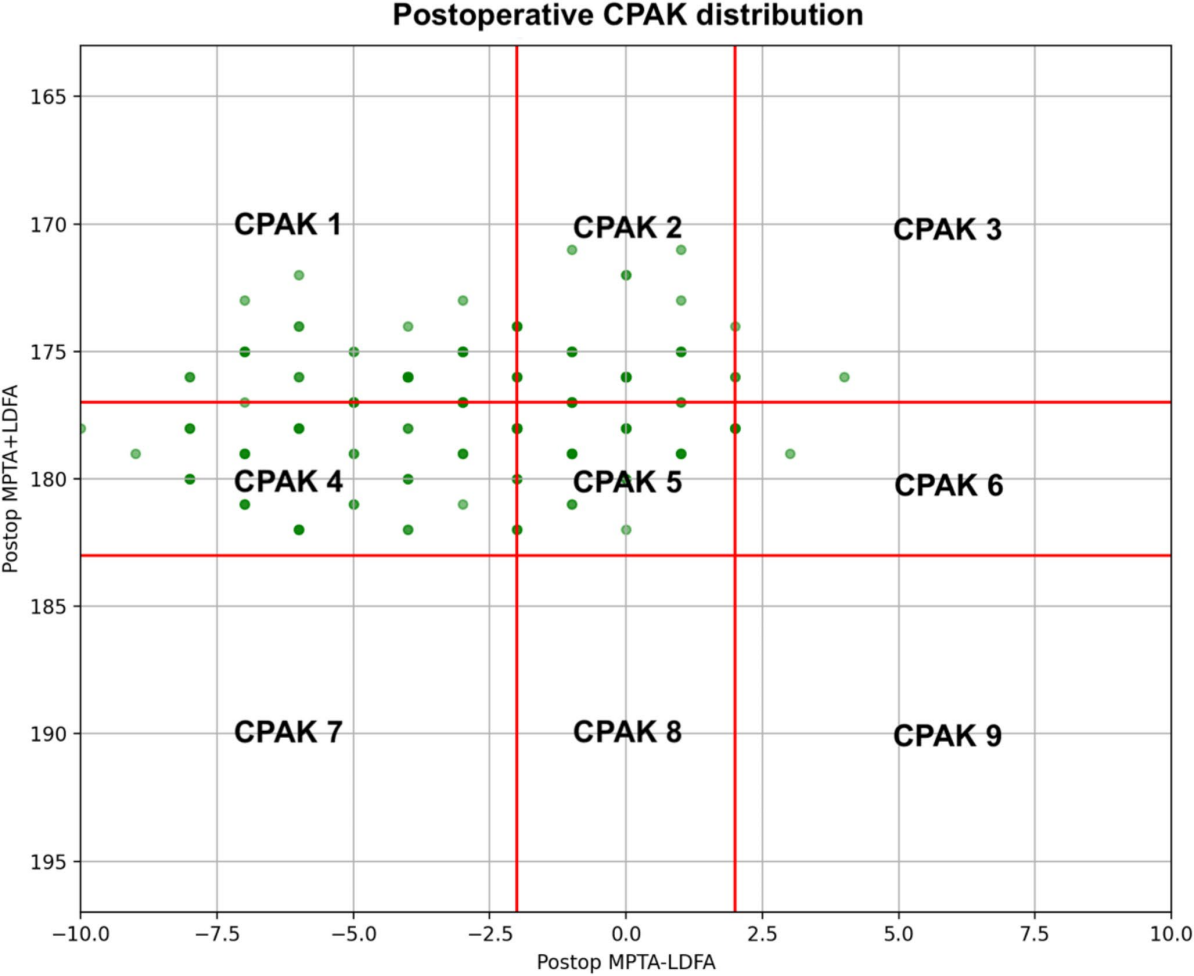
Distribution of patients according to their coronal plane alignment of the knee (CPAK) postoperatively

The most common preoperative CPAK phenotypes were I (*n* = 74, 42.5%) and 2 (*n* = 40, 23.0%), respectively. Among type I phenotypes, 29/74 (39.2%) patients preserved their composition postoperatively, but a noteworthy 36/74 (48.6%) patients converted into type IV. Twenty-six out of 40 Type II knees (65.0%) preserved their alignment, with 10/40 patients (25.0%) shifting to type V. In preoperative types IV and V, 12/16 (75%) and 23/26 (88.5%) patients preserved their original phenotypes, respectively. Almost all valgus categories III, VI and IX were neutralized into phenotypes II and V. CPAK types VII, VIII, and IV were rare throughout this study [Table 1; Figs. 3 and 4].

**Table 1:**
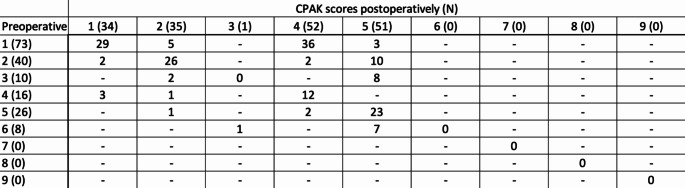
Distribution of coronal alignment of the knee (CPAK) preoperatively, with their consequent distribution postoperatively

## Discussion

The most important finding of this study was that applying the RIKA TKA technique enabled preservation of the native aHKA alignment of the knee while neutralizing the JLO in prevalent varus CPAK phenotypes, but also completely neutralizing valgus knees.

The adoption of robotic assistance in TKA reflects a potential paradigm shift in surgical precision, offering surgeons an advanced tool to enhance intraoperative planning, implant positioning, and overall procedural accuracy, ultimately offering minimization of substantial changes in coronal alignment postoperatively. To our knowledge, this is one of the first studies to incorporate the CPAK classification in pre- and postoperative arthritic knees following a tibia-first robotic RIKA approach.

The distribution of CPAK phenotypes vary significantly in different populations, as shown by Mulpur et al. [21] and Pagan et al. [22] who explored radiological measurements and CPAK phenotypes in arthritic knees across various ethnicities and geographic regions. For example, in Japanese and Indian populations, the prevalence of the type I CPAK phenotype was reported at 53.8% and 58.8%, respectively [21, 23]. This contrasts with the European-Australian population, where only 19.4% exhibited the type I phenotype. Similarly, Sappey-Marinier et al. [10] reported a 33.4% prevalence of the type I phenotype in the French population. A study on Korean populations additionally revealed a high incidence of constitutional varus, indicating significant ethnic variations in knee alignment phenotypes [24]. Consistent with these findings, our study demonstrated that the majority of subjects exhibited the type I phenotype preoperatively. Provided that our cohort primarily comprises individuals of European descent, such prevalence aligns closely with outcomes observed within the French population. These factors highlight the importance of considering population-specific knee phenotypes when planning and evaluating outcomes of RIKA TKA.

In the realm of TKA, various alignment strategies that could influence postoperative outcomes and implant longevity have been developed. Mechanical alignment (MA), rooted as the conventional approach, seeks to align the prosthetic components based on the mechanical axes of the femur and tibia, with the aim of achieving a straight limb (CPAK type V). Conversely, kinematic alignment (KA) described by Howell et al. [25] in 2006, deviates from the traditional paradigm by emphasizing the replication of the patient’s individual joint motion and native anatomy to restore a more physiological knee function. This nuanced approach, generally results in asymmetric bone resections that aims to co-align the axes and joint lines of implants with the three “kinematic” axes of the native joint, which include transverse extension and flexion axes of the tibia and patella, and the longitudinal axis of tibial rotation with respect to the distal femur [15, 25]. The concept of restricted KA integrates elements from both mechanical and kinematic principles, striking a delicate equilibrium [9, 26]. It involves judicious modifications to the natural anatomy while addressing specific deviations that may impact joint stability. One such technique is tibia-first RIKA, which prioritizes tibial alignment as the first surgical step, followed by femoral resections guided by the resulting joint space and soft tissue envelope. The “restricted” element refers to predefined safety limits that constrain excessive deviation from conventional axes to avoid extreme implant positioning. This technique aims to harness the functional benefits of KA while preserving the reproducibility and implant longevity associated with mechanical principles [17]. This can be implemented by multiple methods of conventional instrumentation, computer navigation, personalized instruments, or robotic assistance. Comparisons between KA and MA remain challenging, however. Variability in the grouping of alignment strategies, inconsistent radiographic measurement techniques, and unclear reporting of adverse events have limited the validity of systematic outcome analyses across KA techniques. These issues contribute to confounding conclusions, which compromise reliable insights on clinical outcomes, safety, and efficacy [27, 28].

The findings of our study suggest that preoperative neutral CPAK phenotypes type II and V were preserved postoperatively. This is similar to the findings published by Graichen et al. [17], who tested the capability of restoring morphology and knee phenotypes following a restricted tibia-first, gap-balanced patient-specific alignment technique. In their study, the largest CPAK groups preoperatively were type II, type I and type V, with type II and type V remaining the largest groups postoperatively.

The ability to restore patient JLO postoperatively utilizing the RIKA approach is controversial. In a study by Orsi et al. [29] which compared RIKA and gap-balancing alignment strategies and suggested that RIKA restored a more oblique and more native JLO in extension and flexion compared to gap-balancing. Conversely, Shotaro et al. [30] reported a significant difference between pre- and post-operative CPAK, JLO, and aHKA following kinematic alignment TKA.

In this study, we report a substantial shift from type I preoperative knee to type IV postoperative knee, suggesting changes in patients’ JLO from apex distal to apex neutral, while preserving patients’ aHKA. The highly prevalent postoperative restoration of types IV and V knee in our study also suggests the tendency to neutralize the JLO. This relationship could be attributed to the incorporation of more rigorous gap balancing practices by surgeons in this study aiming to JLO neutralization. However, further research is required to investigate these effects, particularly to elucidate the ability of RIKA approach to restore patient JLO.

With regards to the correction of valgus knees (types II, VI, and IX), current literature supports implementing a MA approach as the gold standard, which seeks to align the prosthetic components with the aim of achieving a straight limb alignment (CPAK type V) [31]. This can be attributed to the reduced stress on medical collateral ligaments and reduction in the risk of secondary knee instability [31]. Consistent with the literature, valgus groups in this study were restored to neutral alignments postoperatively.

With emerging evidence suggesting that CPAK restoration may be associated with more favorable clinical outcomes, Franceschetti et al. [32] further emphasized this correlation by showing that MA TKA does not yield uniform patient-reported outcome measures across different CPAK phenotypes, particularly those diverging from neutral alignment, demonstrating lower satisfaction and functional scores. This highlighted the importance of tailoring alignment strategies to individual phenotypes rather than adhering strictly to MA principles, thereby reinforcing the clinical relevance of CPAK-guided alignment restoration in achieving optimal functional recovery and patient satisfaction.

This prospective study has several limitations that need to be addressed. The primary focus of our study is on assessing coronal alignment exclusively, which presents a limitation by attempting to capture a 3-dimensional knee alignment entity within the confines of a 2-dimensional framework where rotational factors and image quality may impact alignment measurement and subsequent categorization. Nonetheless, conventional X-rays remain the mainstay imaging modality for knee alignment assessment, offering reproducibility and widespread clinical utility. Furthermore, the data focuses solely on radiographic evaluations, without consideration of clinical symptoms or patient-reported outcomes. As a result, the relationship between radiographic changes and their impact on clinically relevant symptoms remains unclear. The last limitation of this study is due to the CPAK classification itself that does not refer to or exclude extra-articular deformities. Coronal alignment measurements and calculations were done as described above explaining outliers in the post-operative group.

## Conclusion

The use of tibia-first RIKA conferred with adequate preservation of the native coronal alignment postoperatively. These findings emphasize the definitions of restricted kinematic alignment as an intermediate between mechanical and kinematic alignment, suggesting that the use of imageless navigation robotic-assisted systems are valid tools for surgeons who seek contemplating tibia-first RIKA in TKA.

## Data Availability

No datasets were generated or analysed during the current study.
